# Computed Tomography Dose in Paediatric Care: Simple Dose Estimation Using Dose Length Product Conversion Coefficients

**DOI:** 10.21315/mjms2018.25.4.8

**Published:** 2018-08-30

**Authors:** Nor Hanani Mohd Tap, Mohamed Ariff Jaafar Sidek, Siti Farizwana Mohd Ridzwan, S. Elavarasi Selvarajah, Faizah Mohd Zaki, Hamzaini Abdul Hamid

**Affiliations:** Department of Radiology, Faculty of Medicine, Universiti Kebangsaan Malaysia Medical Centre, 56000 Cheras, Kuala Lumpur, Malaysia

**Keywords:** simple dose estimation, conversion coefficient, paediatric CT, dose length product, effective dose

## Abstract

**Background:**

The use of multislice computed tomography (MSCT) is increasing worldwide; at the same time, there is a growing awareness of the future risk of cancer associated with greater exposure to radiation. Therefore, there is a need for an accessible method of effective dose estimation. This study aims to estimate the effective doses (EDs) of a variety of paediatric computed tomography (CT) examinations in five age groups using recently published age- and region-specific dose length products (DLPs) as effective dose conversion coefficients.

**Methods:**

A retrospective review was performed over a 12-month period. Patients were assigned to one of five age groups: neonatal, 1-, 5-, 10- and 15-years-old. Age- and region-specific conversion coefficients were applied to the DLP data displayed on the CT console in order to estimate the ED.

**Results:**

Over the 12-month period, there were a total of 283 CT scans, 211 of which were selected for study. The ED estimates for plain CT brain scans in neonatal, 1-, 5-, 10- and 15-yearolds were 2.5, 1.5, 1.4, 1.3 and 0.8 mSv, respectively. For the corresponding CT abdominal scans, the results were 18.8, 12.9, 7.8, 8.6 and 7.5 mSv; these were the highest values recorded. High-resolution CT (HRCT) temporal scans showed EDs of 2.9, 1.8, 1.5 and 1.1 mSv in 1-, 5-, 10- and 15-years-old, respectively. CT scans of the helical thorax had an estimated ED of 4.8, 4.2 and 7.0 mSv in 5-, 10- and 15-years-old, respectively.

**Conclusion:**

An inverse relationship between age and effective dose was demonstrated in CT scans of the brain and abdomen/pelvis. In general, our study showed lower overall EDs compared to other centres.

## Introduction

Multislice computed tomography (MSCT) is valuable for essential imaging modalities. With advances in technology, the use of MSCT in children is increasing worldwide (1). The use of computed tomography (CT) in adults and paediatrics has increased 8-fold since the 1980s, with annual growth estimated to be approximately 10% per year (2, 3). Further, CT contributes approximately 50% of the collective effective dose of radiation from medical imaging in adults, and 11% in children (2). At the same time, there is growing awareness of the potential future risk of cancer associated with the increase in exposure.

The National Academy of Sciences BEIR VII report estimates that an examination of 10 mSv increases the lifetime risk of cancer, both fatal and non-fatal, by approximately 1 in 1000 (4). Children are undoubtedly more radiosensitive than adults are, and they have a longer life expectancy, which results in a larger window of opportunity for the expression of radiation damage. Therefore, radiologists must understand radiation doses and the risks associated with CT to optimise protocol examinations, and communicate effectively with referring physicians.

A variety of CT dose parameters have been used to provide useful information about the dose received by a patient. The most useful parameter currently used is the effective dose (ED). This is defined by the International Commission on Radiological Protection (ICRP) as the sum of the absorbed dose of radiation across all tissues and organs in the body, weighted according to their sensitivity (5, 6). Understanding the effective dose of a radiological examination enables comparisons with other imaging modalities and natural background radiation (6). This information can be useful during discussion with patients, medical staff, ethics committees and research volunteers.

There are many ways to estimate the ED in CT. The gold standard uses Monte Carlo simulation tools with anthropomorphic phantoms. Another method uses ImPACT CT dosimetry software (7). This software uses the scanner model, tube voltage and scan type to select an appropriate Monte Carlo data set. Current CT scanners provide dosage information, including CT dose indices (CTDI_vol_, *mGy*) and dose length product (DLP, *mGy.cm*), for each prescribed scan series. This console-displayed DLP can also be used to estimate the ED when multiplied by an appropriate conversion coefficient derived in medical physics.

In 2008, Thomas and Wang used ED conversion coefficients derived from the old ICRP 60 publication (8, 9). Then in 2010, Deak et al. introduced new age-specific conversion factors, derived from the new ICRP 103, which are more accurate for children of different ages (10).

To our knowledge, no previous studies have been reported in Malaysia using this method to estimate the ED of CT in the paediatric population. This study acts as a baseline for future reference, which can be helpful when developing dose reduction strategies. Thus, the goal of this study is to use these new age-specific conversion factors to estimate the EDs of a variety of paediatric CT examinations at our institution. Specifically, we aim to:

- estimate the ED for a variety of CT paediatric protocols using console-displayed DLP data and age-specific conversion factors;
- determine the EDs of the most frequently performed CT examinations: CT head, CT thorax, CT abdomen/pelvis and high resolution CT (HRCT) temporal scans;
- determine the EDs of less frequent CT examinations: neck/cervical spine, orbit, paranasal sinus (PNS) and three-dimensional (3D) cranial scans; and
- compare the estimated EDs with published paediatric data from around the world.

## Materials and Methods

This is a clinical audit of all the CT scans performed for paediatric patients in our department at Universiti Kebangsaan Malaysia Medical Centre (UKMMC) using a Siemens Somatom Sensation 64-slice scanner. It involves a retrospective review of all the paediatric CT scans that were conducted between January and December 2012.

A total of 211 paediatric patients aged 16 years or less, who were referred to our department for a CT scan and may provide appropriate DLP data according to body region, were included in this study. A further 72 patients were excluded because their DLP data was absent or inappropriately recorded, either by summation across body regions or across individual protocols.

The CT machine used throughout this study was the Siemens Somatom Sensation 64-slice scanner. The basis of the DLP calculations for this scanner was established by an assigned consultant from Siemens Malaysia. For this scanner, the dosimetry methodology is based on the region of CT examination; 16 cm phantom data for all head CT scans and 32 cm phantom data for all body scans, independent of the field of view (FOV) and patient age (11, 12).

Therefore, the paediatric body DLP values that were calculated based on the adult 32 cm phantom had to be multiplied by a factor of two, before the age-appropriate conversion coefficient was applied (8, 11, 13, 14). This gave a similar value to the DLP estimate based on the 16 cm phantom. Conversion coefficients for paediatric head examinations were based on a 16 cm phantom, as for adults. Thus, the console-displayed DLP values did not require an additional multiplication factor. This was further confirmed by the CT scanner vendor.

Paediatric patients were considered to be those up to 16 years old, following the general guidelines for the age of paediatric patients (12). Patients were assigned to five age groups for the application of age-specific conversion coefficients: newborn to 3 months (0 years), 4 months to 2 years 11 months (1 year), 3 years to 7 years 11 months (5 years), 8 years to 14 years 11 months (10 years) and 15 years and older (adult).

Patient scan details had been previously recorded in the departmental CT logbook. The following data were collected: patient age, examination protocol (anatomical regions, single/dual phase) and console-displayed DLP values. For multiregion CT examinations, each region-specific DLP was recorded. For example, chest/abdomen/pelvis CT scans were performed as a single examination without anatomical overlap, but with a transition in the tube current at the dome of the liver. Two separate DLP values were generated by the scanner software for the chest and abdomen/pelvis components.

Similarly, if two separate protocols were performed involving the same body region (for example, CT brain plain followed by contrast) then the DLP values for the two components were recorded separately, enabling dose data for each protocol to be assessed. The CT protocols and scanning parameters recommended by the Siemens guidelines were followed accordingly (15). Scans were reviewed on the PACS system if there was any uncertainty regarding the scan protocol, or if significant deviation from our standard protocols was suspected.

Data were recorded in a Microsoft Excel spreadsheet. The data collection represented a non-random sampling of all the records gathered over a designated period. We provided the mean and standard deviation of the DLPs and EDs that were calculated for each age group and respective standard protocol.

## Results

This retrospective review of all paediatric CT examinations yielded information on a total of 283 CT protocols performed. In 42 cases, the DLP data were not recorded, and 27 cases were excluded as they were recorded as a summation across a bodily region. Additionally, three other cases of CT extremities were excluded, as there were insufficient appropriate extremity conversion coefficients.

The complete DLP data available for 211 protocols consisted of 93 neuroradiology and 118 body scans. The patients’ ages ranged from 1 day to 16 years, with a mean of 8.3 years.

### Age and Frequency Distribution

The age distribution of the paediatric CT examinations showed that the 10-years-old group (from 8 years to 14 years and 11 months) accounted for 44% of the total examination. As expected, the fewest examinations were done for the 0-years-old group (newborn to 3 months).

The majority of the examinations were CT brain (49%) scans, followed by CT temporal (14%), CT thorax (11%), CT abdomen (9%) and CT spine (6%) scans. Less frequent CT examinations, which account for the remaining 11%, included CT neck, CT extremities, CT orbit and CT thorax/abdomen/pelvis scans.

### Types of CT Scan with DLP and ED Data

We concentrated on four major CT examinations that were frequent in this population when describing the DLP and ED data: CT brain (plain), abdomen/pelvis, HRCT temporal and CT helical thorax scans. We do not describe the DLP and ED of the less frequent CT examinations in this paper.

The most frequently performed CT examinations are CT brain scans, followed by high resolution CT (HRCT) scans of the temporal region and abdomen/pelvis and CT scans of the thorax. Table 1 shows the mean and standard deviation of the DLP, and the calculated ED estimates for each age group. There were no patients in the 0-years-old group for HRCT temporal scans or the 0- and 15-years-old groups for CT helical thorax scans. Only one patient in the 1-year-old group had a CT thorax scan, thus the standard deviation could not be calculated.

The mean estimated ED for plain CT brain scans ranged from 2.5 mSv in a neonate to 0.8 mSv in a 15-year-old. As expected, the plain and contrasted CT brain scan was associated with double the effective dose of the plain CT brain scan. The ED for CT temporal scans ranged from 2.9 mSv in a 1-year-old to 1.1 mSv in a 15-year-old. The CT abdomen/pelvis scans showed a wide range of EDs from 18.8 mSv in a neonate to 7.5 mSv in a 15-year-old.

**Figure 1:**
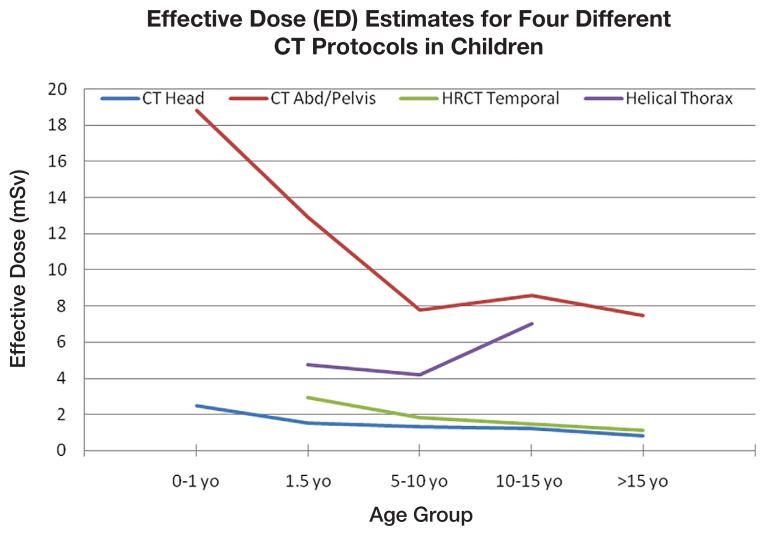
Estimation of ED for four different CT protocols in children

There was an inverse proportional relationship between age and ED for CT brain, HRCT temporal and CT abdominal scans (Figure 1). On the contrary, there was no clear relationship between age and ED for CT thorax scans.

**Figure 2:**
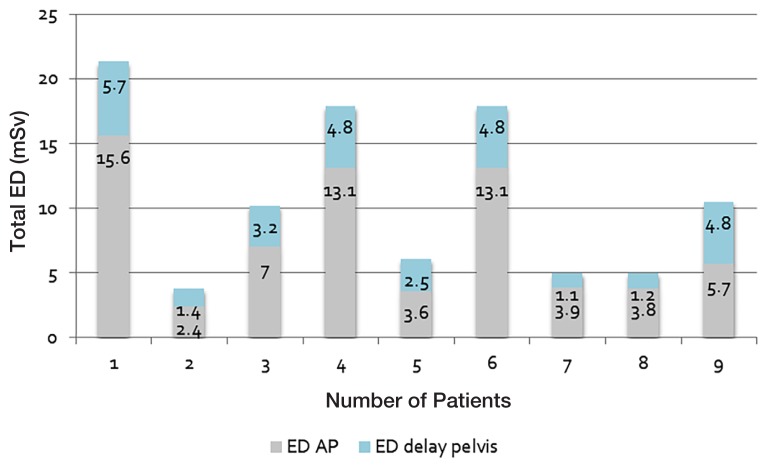
Additional EDs for delay scans in the pelvis during CT abdominal examinations

**Figure 3:**
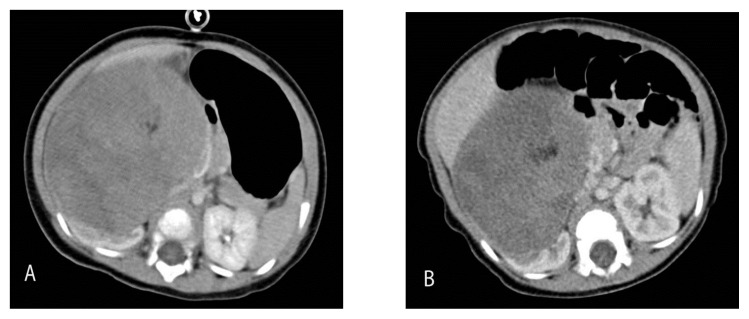
CT abdominal scan of a 14-month-old child: (A) at the time of diagnosis using 100 kVp/100 mAs and (B) at 4 months follow-up using 100 kVp/50 mAs. The calculated EDs for these examinations were: (A) 12.9 mSv and (B) 6.5 mSv. With the lower effective charge, the image remains diagnostic, even though it is grainier, and most importantly it resulted in significant dose reduction

We found that a few of the CT abdomen/ pelvis examinations included a delay scan in the pelvic region. This caused a significant increase in the ED measurement. During this study period, 9 of the 25 patients who had CT abdomen/pelvis examinations had a delay scan in the pelvis. The additional EDs were calculated and they varied from 1.1 to 5.7 mSv (Figure 2). An example of a case with a different CT dose performed at two different times is described in Figure 3.

## Discussion

The console-displayed DLP values are estimates based on standardised dose measurements. The weighted CT dose index (CTDI_w_) is identified by the manufacturer for each scanner model using polymethylmethacrylate cylinder phantoms of standardised sizes, usually a 16 cm diameter head phantom and a 32 cm body or torso phantom. The scanner software uses these data, in association with technical parameters for an individual scan and the length of coverage prescribed by the radiographers, to generate a DLP estimate (6–10, 15).

Effective dose (ED) estimates can be derived from the DLP using appropriately normalised coefficients; E = E_DLP_ × DLP, where E_DLP_ is a region-specific dose conversion coefficient expressing the effective dose normalised to the DLP in a standard CT dosimetry phantom (mSv. mGy^−1^.cm^−1^) (16).

Shrimpton et al. derived an age and region-specific DLP for ED conversion coefficients using Monte-Carlo simulations in a family of mathematical phantoms (neonatal, 1-, 5- and 10-years-old) (13). These coefficients are used in children of various ages for head, neck, chest and abdomen/pelvis examinations. The Monte-Carlo computations were based on three models of scanners, representing the first generation of CT scanners, and the conversion factors were computed based on the old ICRP 60 recommendations (5).

There is an important difference between children and adults in CT dosimetry; the energy imparted to the centre of a smaller paediatric body is higher than that imparted to the centre of a larger adult body for the same exposure. Consequently, the organ doses and EDs are higher in children than in adults, and the younger the child, the greater the increase (17). Studies in medical physics literature consider the 16 cm phantom to be a closer approximation to paediatric chest and abdomen size than the 32 cm phantom for paediatric body dosimetry. There is less disparity in the size of paediatric and adult heads, so it is generally considered acceptable to use the 16 cm phantom for head CT scans at all ages (13, 14).

There is a wide range of EDs in paediatric CT scans across all age groups. Low dose protocols mainly pertain to head and facial bone CT scans, while high dose protocols pertain to CT scans of the abdomen and pelvis. The higher organ doses in younger children mean that the conversion coefficients for a neonate are approximately five times greater for the head, and three times greater for the body, than for a 15-year-old (8).

Our study demonstrated an inverse relationship between the ED and age for CT brain and CT abdominal scans, similar to the Canada study (9). The results of the CT thorax scans were similarly variable, with no clear relationship to age. However, there was not enough data on CT thorax scans, as we did not find any complete DLP data for age groups 0 and 15.

Adding a delay scan in the abdomen/pelvis during examination protocols has been proven to increase the ED by 30%–50% in paediatric patients. Two of the nine-delay pelvis scans were conducted as ovarian cancer follow-up scans. The delay pelvis scan was done only once for a new case of ovarian teratoma in a 15-year-old girl. The other five cases were done for follow up and staging of hepatoblastoma, nephroblastoma or osteosarcoma.

Even though the objective of performing a delayed pelvis phase is to enhance visualisation of the pelvic structures, this phase has been omitted since this study and is reserved for trauma cases with suspicion of urinary bladder injury. We also advocated for a single-phase CT abdomen/pelvis scan in all paediatric patients. When there is any doubt regarding further characterisation of liver or kidney lesions, which require multiple phase CT imaging in adults, the patient will be sent for magnetic resonance imaging (MRI) which does not impose a radiation burden.

### Comparison with Other Centres

#### CT brain

**Figure 4:**
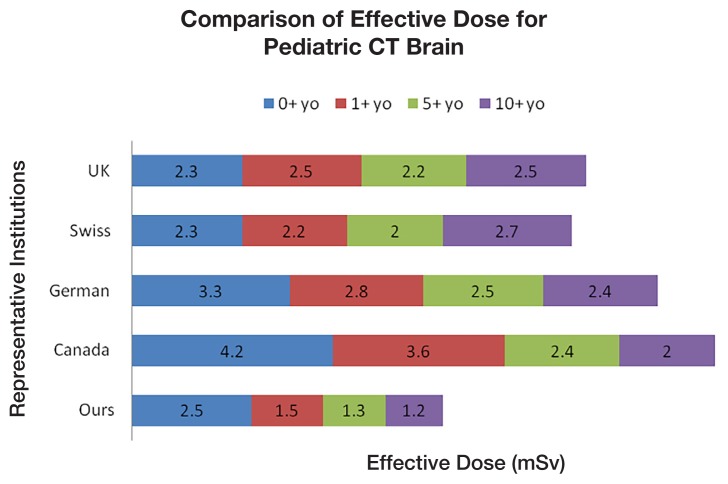
Comparison of the effective doses for paediatric CT brain scans in our study and representative institutions

CT brain scans showed an inverse proportional relationship between age and ED, a similar trend to that shown in the Canadian and German studies (9, 18). The results (Table 2) showed that the mean ED for ages 1, 5 and 10 were the lowest among the centres (Figure 4). For the 0-year-old group, the ED was slightly higher than the Swiss and UK series (13, 19).

#### CT abdomen pelvis

The calculated mean ED for CT scans of the abdomen and pelvis showed that the highest dose was in age group 0 (newborn up to 3 months old) which was estimated to be 18.8 mSv. The ED for this group ranged from 17.4 to 20.3 mSv. For this age group, the recommended CT protocol was a spiral mode, using 80 kVp, and an effective charge of 33 mAs. Instead, we used a CT protocol of 100 kVp and effective charge of 100 mAs. The resultant ED was the highest among the centres, 13.1, 11.7, 10.5 and 13.7 mSv for the Canadian, German, Swiss and UK studies, respectively (9, 13, 18, 19).

In one case, follow-up for a right nephroblastoma, we used two different CT protocols as illustrated in Figure 3. The first CT was conducted when the child was 14 months old using 100 kVp/100 mAs; the follow-up was approximately four months later using a lower dose exposure of 100 kVp/50 mAs. The calculated EDs for these examinations were 12.9 and 6.5 mSv, respectively. This shows a significant reduction in the ED by almost 50%, and illustrates how important it is to adapt the dose setting according to age and body weight.

Given that CT scans of the abdomen and pelvis showed a higher ED than other CT protocols, we decided to examine the CT parameters for each case. Surprisingly, we observed that the majority of the cases did not follow the CT parameters recommended by Siemens. For example, for the 0-year-old group (newborn to 3 months), 100 mAs/100 kV was used instead of 33 mAs/80 kV. The average ED for this group was 18.8 mSv. The rest of the age groups showed relatively low EDs compared to other centres. However, there were unnecessary additional EDs from delay pelvis scans.

#### CT thorax

There are no data for 0- and 15-year-old groups. The rest of the calculated EDs were lower than in other studies, except for the 10-year-old group. Our EDs were higher than the values derived by Thomas et al. in Canada, as well as Galanski et al. in Germany (9, 19). To ensure that paediatric CT examinations are carried out in a dose-optimised fashion, Galanski et al. recommended dose adaptation of the effective charge setting (18). Table 2 summarises a comparison of the ED in our study and those by the aforementioned institutions.

Based on our results, we devised institutional guidelines for radiation exposure based on the as low as reasonably achievable (ALARA) principle with reference to the American College of Radiology (ACR) guidelines and previous literature to reduce the radiation dose in children (20, 21). We also stressed the exclusion of the delayed pelvis phase from routine CT scans of the abdomen and pelvis except for cases related to urinary bladder injury. We hope to achieve optimum CT examination among paediatric patients with low dose radiation exposure, without compromising the diagnostic quality of the images.

Since the completion of this study, DLP data for each scan series has been automatically transferred to the picture archiving and communication system (PACS). In the future, we hope that the DLP values for multi-region examinations will be recorded separately. The DLP data that are summated across anatomical regions are not meaningful for use in this method of effective dose estimation. In order to ensure that paediatric CT examinations are carried out in a dose-optimised protocol, the CT protocols recommended by Siemens should be used, knowing that there has been increased radiation dose awareness among CT scanner manufacturers (21).

### Limitation and Suggestion

Our study had several limitations. The most important limitation is that we only have a small sample compared to other centres, where studies were conducted at a national paediatric centre or as a cumulative study over several hospitals. The DLP data were estimates generated by scanner software, which depend on scan parameters. We found that the DLP derived EDs are 14%–37% lower than the phantom derived values (22). We do not have gold standard direct dosimetry data to compare. Perhaps, the next step is to compare our data with ED estimates using an anthropomorphic phantom. Nevertheless, in our search for accuracy, this study is an estimate of EDs and, more importantly, the values can be used for comparative assessment and promotion of dose-reduction strategies.

## Conclusion

Despite the limitations discussed, we believe that age- and region-specific DLP conversion coefficients provide an accessible and user-friendly method for ED estimation. An inverse relationship between age and ED was demonstrated in CT brain and CT abdomen/ pelvis scans. Our study displayed, generally and comparatively, lower overall ED estimation compared to other centres. We hope that these data can be used to establish institutional dose estimates, and to develop dose reduction strategies in the future.

## Figures and Tables

**Table 1:** DLP and effective dose estimates for CT head, abdomen/pelvis, HRCT temporal and CT thorax

CT examinations	Patients		No. of patients (*n*)	DLP (mGy cm)		ED (mSv)	
		
	Total patients (*N*)	Age (years)		Mean	SD	Mean	SD
Head (P)	118	0	28	289	31.2	2.5	0.26
		1	13	290	94.4	1.5	0.49
		5	12	383	75.5	1.3	0.27
		10	43	495	144.9	1.2	0.39
		15	22	422	91.3	0.8	0.16

Abdomen/Pelvis	25	0	3	234	25.4	18.8	2.01
		1	4	230	74.2	12.9	5.58
		5	7	216	92.5	7.8	2.81
		10	6	303	168.5	8.6	5.79
		15	5	418	44.0	7.5	2.37

HRCT temporal	30	0	0	0	0	0	0
		1	16	551	114.8	2.92	0.61
		5	7	526	256.1	1.79	0.89
		10	5	543	171.3	1.47	0.46
		15	2	579	NA	1.10	0.00

Helical thorax	24	0	0	0	0	0	0
		1	1	102	NA	4.76	NA
		5	13	137	80.6	4.20	3.21
		10	10	310	72.5	7.02	1.71
		15	0	0	0	0	0

**Table 2:** Estimated dose for CT brain, CT abdomen/pelvis and CT thorax in this study compared to other centres

CT examination	Age group	Quantity	Ours	Canada (8)	German (18)	Swiss (19)	UK (13)
(a) CT brain	0	DLP	290	490	390	270	270
		ED	2.5	4.2	3.3	2.3	2.3
	1	DLP	290	680	520	420	470
		ED	1.5	3.6	2.8	2.2	2.5
	5	DLP	380	690	710	560	620
		ED	1.3	2.4	2.5	2.0	2.2
	10	DLP	500	740	920	1000	930
		ED	1.2	2.0	2.4	2.7	2.5
	15	DLP	420	730	NA	NA	NA
		ED	0.8	1.4	NA	NA	NA

(b) CT abdomen/ pelvis	0	DLP	230	268	145	130	170
		ED	18.8	13.1	11.7	10.5	13.7
	1	DLP	240	370	255	300	250
		ED	12.9	11.1	13.1	15.4	12.9
	5	DLP	215	420	475	380	500
		ED	7.8	8.4	16.6	13.3	17.5
	10	DLP	303	595	500	500	560
		ED	8.6	8.9	12.3	12.3	13.8
	15	DLP	420	395	NA	NA	NA
		ED	7.5	5.9	NA	NA	NA

(c) CT thorax	0	DLP	–	40	55	110	200
		ED	–	2.8	3.9	7.8	14.1
	1	DLP	102	73	110	200	230
		ED	4.8	3.4	5.1	9.3	10.7
	5	DLP	140	118	210	220	370
		ED	4.2	3.7	6.6	6.9	11.6
	10	DLP	310	175	205	460	580
		ED	7.0	4.1	4.8	10.8	13.6
	15	DLP	–	193	NA	NA	NA
		ED	–	2.8	NA	NA	NA

## References

[1] Muhogora WE, Ahmed NA, AlSuwaidi JS, Beganovic A, Ciraj-Bjelac O, Gershan V (2010). Paediatric CT examinations in 19 developing countries: frequency and radiation dose. Radiat Prot Dosim.

[2] Brenner DJ, Hall EJ (2007). Computed tomography— an increasing source of radiation exposure. N Eng J Med.

[3] Mettler FA, Bhargavan M, Faulkner K, Gilley DB, Gray JE, Ibbott GS (2009). Radiologic and nuclear medicine studies in the United States and worldwide: frequency, radiation dose and comparison with other radiation sources-1950–2007. Radiology.

[4] Committee to Assess Health Risks from Exposure to Low Levels of Ionizing Radiation (2006). Health risks from exposure to low levels of ionizing radiation: BEIR VII Phase 2 [Internet].

[5] ICRP (1991). 1990 Recommendations of the International Commission on Radiological Protection ICRP Publication 60.

[6] McCollough CH, Schueler BA (2000). Educational treatise: calculation of effective dose. Med Phys.

[7] ImPACT (Imaging Performance Assessment of CT scanners) (1998). Type testing of CT scanners: methods and methodology for assessing imaging performance and dosimetry. [Internet].

[8] Shrimpton PC, Wall BF (1992). Assessment of patient dose from computed tomography. Radiat Prot Dosim.

[9] Thomas KE, Wang B (2008). Age-specific effective doses for pediatric MSCT examinations at a large children’s hospital using DLP conversion coefficients: a simple estimation method. Pediatric Radiology.

[10] Deak PD, Smal Y, Kalender WA (2010). Multisection CT protocols: sex-and age-specific conversion factors used to determine effective dose from dose-length product. Radiology.

[11] Ulzheimer S, Leidecker C, Endt H (2011). Dose parameters and advanced dose management on SOMATOM scanners.

[12] Reider-Demer M, Zielinski T, Carvajal S, Anulao K, Van Roeyen L (2008). When is a pediatric patient no longer a pediatric patient?. J Pediatr Health Care.

[13] Shrimpton PC, Hillier MC, Lewis MA, Dunn M (2005). Doses from computed tomography (CT) examinations in the UK-2003 review.

[14] Chapple CL, Willis S, Frame J (2001). Effective dose in paediatric computed tomography. Phys Med Biol.

[15] Bredenhöller C, Feuerlein U (2002–2004). Somatom Sensation 64 application guide: protocols, principles and helpful hints. Software version *syngo* CT 2005A.

[16] Bongartz G, Golding SJ, Jurik AG, Leonardi M, van Persijn van Meerten E, Rodríguez R (2004). CT safety and efficacy: a broad perspective European Guidelines for Multislice Computed Tomography [Internet].

[17] Huda W (2002). Effective doses to adult and pediatric patients. Pediatr Radiol.

[18] Galanski M, Nagel HD, Stamm G (2006). Paediatric CT exposure practice in the Federal Republic of Germany. Results of a nation-wide survey in 2005/06 [Internet].

[19] Verdun FR, Gutierrez D, Vader JP, Aroua A, Alamo-Maestre LT, Bochud F (2008). CT radiation dose in children: a survey to establish age-based diagnostic reference levels in Switzerland. Eur Radiol.

[20] The American College of Radiology (2016). ACR-ASERS-CRT- MR-SPR practice parameter for the performance of pediatric computed tomography (CT) of the abdomen and computed tomography (CT) of the pelvis. [Internet].

[21] Zacharias C, Alessio AM, Otto RK, Iyer RS, Philips GS, Swanson JO (2013). Pediatric CT: strategies to lower radiation dose. AJR Am J Roentgenol.

[22] Chapple CL, Willis S, Frame J (2001). Effective doses in pediatric computed tomography. Phys Med Biol.

